# An evaluation model for automobile intelligent cockpit comfort based on improved combination weighting-cloud model

**DOI:** 10.1371/journal.pone.0282602

**Published:** 2023-03-03

**Authors:** Jianjun Yang, Qilin Wan, Jiahao Han, Shanshan Xing

**Affiliations:** School of Automobile and Transportation, Xihua University, Chengdu, China; Al Mansour University College-Baghdad-Iraq, IRAQ

## Abstract

Aiming at the comfort evaluation of automobile intelligent cockpit, an evaluation model based on improved combination weighting-cloud model is established. By consulting relevant literature, 4 first-class indexes and 15 second-class indexes, including noise and vibration, light environment, thermal environment and human-computer interaction, are selected to establish a comfort evaluation system. Later the subjective and objective weights obtained by improved Analytic Hierarchy Process (AHP) and Technique for Order Preference by Similarity to an Ideal Solution (TOPSIS) are combined by Game Theory. Considering the fuzziness and randomness of the index system, the combination weights obtained by Game Theory are combined with the cloud model. The floating cloud algorithms is used to determine the first-class and second-class index clouds and the comprehensive evaluation cloud parameters. Improvements were made in two commonly used similarity calculation methods, the expectation curve method (ECM) and the maximum boundary curve method (MCM). A new similarity calculation method is defined to optimize the evaluation results and determine the final comfort evaluation grade. Lastly, a 2021 Audi intelligent car under a certain working condition was selected to verify the correctness and rationality of the model using the fuzzy evaluation method. The results show that the cockpit comfort evaluation model based on the improved combination weighting-cloud model can better reflect the comprehensive comfort of automobile cockpit.

## Introduction

With the progress of science and technology, the automobile has begun to develop towards the path of intelligence and comfort. Various industries have conducted in-depth studies on comfort over the years, but none of them have been able to give a clear and universal definition. One of the important reasons is the difference between individuals in understanding the concept of comfort and discomfort. According to Wechsler’s dictionary, comfort is considered to be a state of relaxation, pleasure and subjective feeling [1]. Slater’s definition of comfort is more in line with the automobile cockpit perspective. Slater considers comfort as a state of physical, mental and physiological harmony between a person and the environment [2, 3]. Here it can be understood as a state of harmony between the driver or occupants of the cockpit external environment and themselves. This state is directly reflected in the human perception and is judged by the human body on the level of comfort.

The comfort of the automobile cockpit largely affects the psychological changes of the driver and unconsciously alters his driving state [4, 5]. In order to ensure the safety of the car driving process, it is necessary to study the comfort of the automobile cockpit. In recent years, more and more scholars have started to conduct research on cockpit comfort. For example, in the aspect of vehicle interior noise [6], established a model to evaluate the overall comfort of vehicles by using the specific index method. In order to estimate the change of noise quality [7], used subjective noise control system to analyze the sound quality of real multi-channel noise controller. In light environment research, an evaluation model of cockpit light environment comfort was proposed by [8, 9] found that using appropriate curve light parameters can reduce the glare emitted by adaptive curve lights, and thus designed the adaptive headlamp of intelligent vehicles. In the context of thermal environment [10], studied the influence of the interaction between window clearance and air conditioner on thermal comfort [11]. Summarized the evaluation models of automobile thermal comfort. In terms of human-computer interaction [12], proposed to study the active seat model to improve the perceived comfort and activity level of vehicle passengers. It is worth noting that Ene, A et al. put the thermal comfort and acoustic comfort in the carriage together for research [13]. It has a great directional effect on the research method of this paper. The experiment confirmed the high correlation and mutual influence between the thermal comfort and the acoustic comfort in the carriage, thus confirming the correctness of the research. It is clear from the above that most of the current scholars mainly study automobile cockpit from the aspects of interior noise, automobile vibration, light environment and thermal environment.

Although the research direction of intelligent cockpit covers a wide range, it is mainly based on environmental indexes such as noise, light, heat, etc. And the established models are mostly aimed at a single index. Therefore, this paper builds on the ideas of the previous paper to study the relationship between the comprehensive index and the automobile intelligent cockpit. In order to feed back the results better, this paper uses the comfort value to reflect the relationship between automobile cockpit, single index and comprehensive index. The grades of comfort are quantified into the corresponding intervals, and experts can score the comfort grade of intelligent cockpit according to the standard. Most evaluation methods can be applied for the problem of cockpit comfort evaluation. For example, the subjective weighting can be done by AHP [14], fuzzy comprehensive evaluation [15], grey correlational analysis, etc. The objective weighting can be done by entropy method [16], factor analysis, TOPSIS, etc. Most of the traditional weighting methods have complicated calculation steps and low operation efficiency. The improved subjective weighting method and objective weighting method can achieve the purpose of simplifying the computational steps and improving the operational efficiency. According to the literature, the combination weight of Game Theory is an algorithm that can combine the advantages of different algorithms [17]. Its basic idea is to use Game to combine different weights with each other and finally get a combination weight between multiple weights.

Owing to the fuzziness and randomness of the evaluation indexes, it is difficult to reflect the actual situation completely and correctly only by using the method of weighting. Checking the literature review, the cloud model can well explain the fuzziness and randomness of indexes [18]. Each index corresponds to a set of evaluation clouds respectively, and a set of standard index clouds is defined the comfort level. When the evaluation model is established, the evaluation cloud corresponding to the index is compared with the standard cloud in turn. If there are intersecting parts between two cloud models, the degree of intersection between the two cloud models can be quantified by using the functional relationship. At present, the commonly used solutions are ECM and MCM proposed by Li Hailin [19]. But the former does not consider the influence of a certain parameter of the cloud thickness in the local boundary conditions. And the latter overemphasizes the role of a certain parameter, which makes this method have some defects.

This paper combines evaluation class algorithm, cloud model and automobile cockpit comfort to provide an alternative approach for studying cockpit comfort models. The results show that the evaluation model based on the improved combination weighting-cloud model accomplishes the task of evaluating the automobile cockpit comfort very well. Compared with the method using fuzzy evaluation, the model can better reflect the real working conditions of the automobile cockpit. The choice of improved AHP and TOPSIS greatly simplifies the computational steps. The combination weighting method based on Game Theory better neutralizes the extremes and deficiencies of subjective and objective. Finally, the evaluation system established in this paper based on four indexes has certain reference significance for future research on the comfort of automobile cockpits.

## Research methodology

This study was conducted in December 2021, and the site for the experiment was chosen on the campus of Xihua University. The experimental equipment was a 2021 Audi A6 car. We collaborated with 8 researchers from the university and company. Written consent was obtained from 8 researchers for this experiment. All eight researchers signed a “Participant Informed Consent Form” and volunteered to participate in the study. All eight researchers participated with us throughout the experiment. Our study was approved by the Automotive and Transportation Ethics Committee of Xihua University. The approval number is 2021LL(01). My team and I were informed and started to conduct the study. The experimental study was conducted on the highway near the 8th academic building of Xihua University.

All participants were 18 years of age or older, and information about the test subjects can be found in the "Participant Informed Consent Form" attached. A total of eight experts in the field of automobile research between the age of 38–56 were selected for the study, including four females and four males. Due to the limited seating capacity of the car cockpit, all 8 experts were scored separately for the same operating conditions of the cockpit comfort during the actual study. The whole process of the experiment was open and transparent, and no malpractice occurred. The experts’ scores for a given index are organized as data in the tables that follow.

### Intelligent cockpit evaluation system

**Fig 1:**
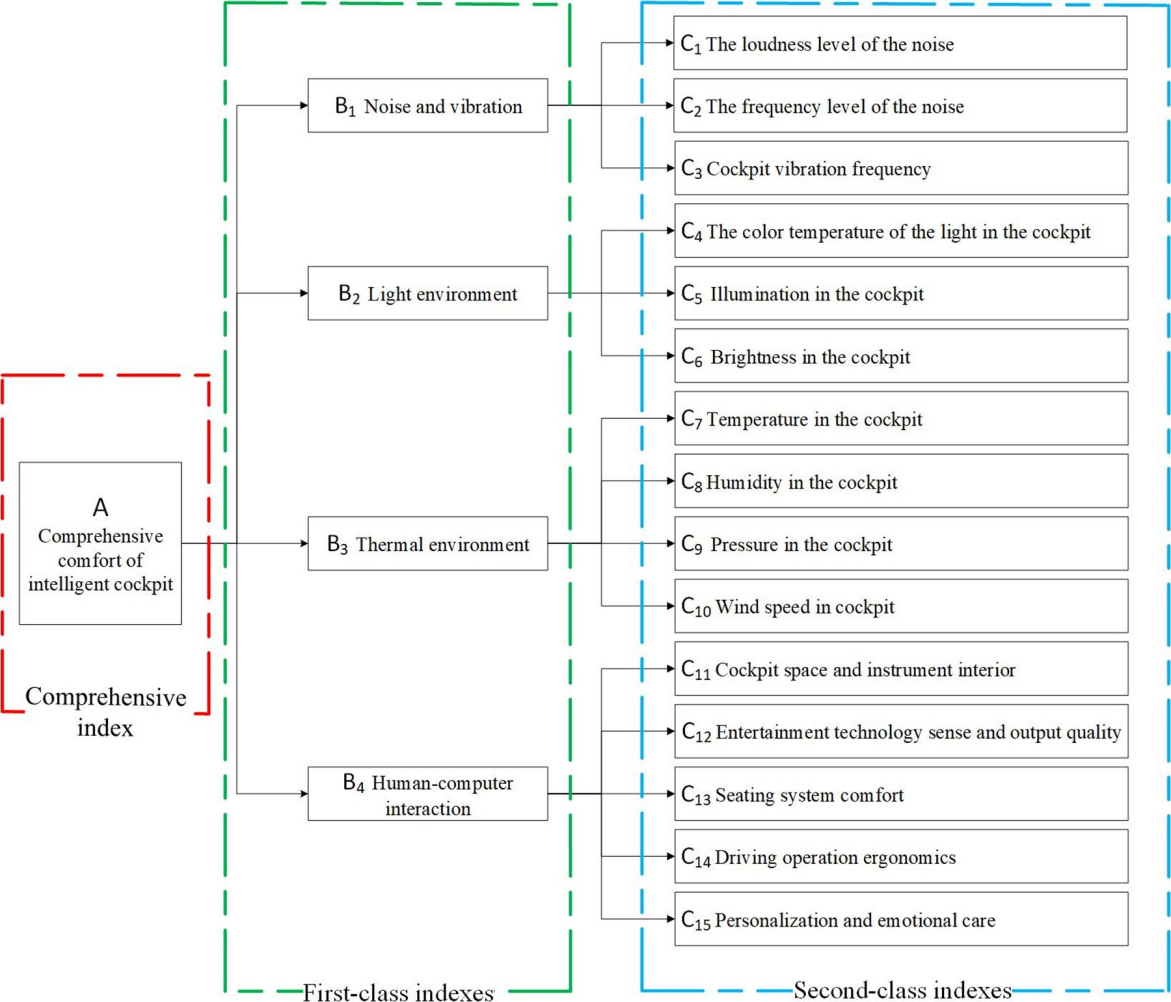
Evaluation system of comfort indexes of intelligent cockpit. The cockpit system consists of three parts. The content in the red box represents the comprehensive index. The content in the green box represents the first-class index. The content in the blue box represents the second-class index.

When establishing the evaluation system of intelligent cockpit of automobile, the external environmental indexes and the internal indexes of automobile should be considered comprehensively. After reviewing relevant literature [20–24] and listening to experts’ opinions, noise and vibration, light environment, thermal environment and human-computer interaction was selected as the first-class indexes in this paper. At the same time, 15 second-class indexes, such as noise loudness, illumination in the cockpit and temperature in the cockpit were selected. Thus, the comfort evaluation index system of automobile intelligent cockpit was established, as shown in Fig 1. The comprehensive evaluation index in the figure reflect the influence of its comprehensive environment on the cockpit comfort condition.

### Comfort standards

International research on comfort started earlier, and the standard of comfort has been improving with the times. At present, the ISO standard is often referred to. In 1970, Professor Fanger proposed the PMV-PPD index with reference to the ISO7730 standard, and used this index to complete the determination and analysis of thermal comfort [25]. At the same time, Professor Fanger also graded the predicted mean vote PMV and evaluated the comfort in seven stages from -3 (cold sensation) to 0 (intermediate sensation) to +3 (hot sensation) [26, 27]. In 2001, the manikin-based equivalent temperature (*T*_*eq*_) of a uniformly heated environment was proposed in SAEJ2234-2001 [28]. The assessment standard based on the transport thermal environment refer to ISO 14505-2-2006. It provides more explanation and calculation of the evaluation index *T*_*eq*_ [29]. Checking the relevant comfort standards, it can be found that the existing evaluation standards for automobile cockpit comfort are based on the thermal environment. Besides, the evaluation standards have the characteristics of unity.

Taken together, this paper extends the study of the comfort of automobile cockpits to four indexes: noise and vibration, acoustic environment, thermal environment and human-computer interaction. A single evaluation standard for thermal environment cannot be specific and adapted to the comprehensive environment of the automobile cockpit. With reference to the automobile industry evaluation standard, the evaluation results were divided into five grades (Ⅰ-Ⅴ) [30]. Combined with the scoring habits of the testers, the length of the interval for each grade was expanded from 1 to 2. This was done to improve the match between the evaluation scale and the test results. The numbers 0 to 10 were chosen to quantify the evaluation results, where the numbers 0 and 10 represent the two poles of the comfort evaluation result respectively. The number 0 is unbearable and the number 10 is very comfortable. The numbers in between can determine the evaluation results according to the intervals divided between the grades. The corresponding intervals and descriptions of the five grades are shown in Table 1.

**Table 1:** Evaluation grade of comfort.

Grade	Ⅰ	Ⅱ	Ⅲ	Ⅳ	Ⅴ
**Score interval**	[0,2]	[2,4]	[4,6]	[6,8]	[8,10]
**Description**	Unbearable	Very uncomfortable	Uncomfortable	Slightly uncomfortable	Comfortable

There are 5 score intervals of comfort, each of which is 2 in length and the total length of the interval is 10. Experts score each index according to the comfort level of the automobile cockpit, with the score controlled to one decimal point.

### Evaluation model based on improved combination weighting-cloud model

This section introduces the principle of combination weighting in Game Theory. Firstly, the subjective weights are obtained by improved AHP, and the objective weights are obtained by improved TOPSIS. Secondly, the subjective weights and objective weights are combined by the principle of Game Theory, and finally the combination weights based on Game Theory is obtained.

#### Improved AHP

AHP was first put forward by American Professor Saaty.T.L in the early 1970s. It was an analytic method of hierarchical weight decision for applying network system theory. Also it was a multi-objective comprehensive evaluation method. AHP is a qualitative and quantitative decision analysis method [31]. Due to its strong subjectivity, it is often used to calculate subjective weight. The traditional AHP has tedious calculation tasks, and consistency test is required in every calculation. Thus, it limits the number of judgment matrices. Therefore, the improved AHP [32] is chosen to calculate the subjective weight in this paper. The steps are as follows:

Step 1. Construct a judgment matrix *A*:

$$A={\left(a_{ij}\right)}_{n\times n}(i,j=1,2,3\cdots n)$$ (1)

where *a*_*ij*_ is the importance degree of the *i*-th index compared with the *j*-th index. In the same way, *a*_*ji*_ is the importance of the *j*-th index compared with the *i*-th index. It satisfies *a*_*ij*_ = 1/*a*_*ji*_, *a*_*ij*_>0, *a*_*ii*_ = 0. In this paper, "Proportional Relative Formula" (*W*_*i*_/*W*_*j*_), inspired by AHP theory, is used to assign value to *a*_*ij*_. *W*_*i*_ and *W*_*j*_ respectively represent the relative distribution values of the two indexes that are compared with each other, and *W*_*i*_+*W*_*j*_ = 1 is satisfied.

Step 2. Let *b*_*ij*_ = lg *a*_*ij*_, get the antisymmetric matrix *B* of the judgment matrix *A*, where *B* = (*b*_*ij*_)_*n*×*n*_ (*i*, *j* = 1,2,3⋯*n*) and satisfy *b*_*ij*_ = −*b*_*ji*_.

Step 3. Let *b*_*ij*_ transform as follows to get *c*_*ij*_:

$$c_{ij}=\frac{1}{n}\sum_{k=1}^{n}\left(b_{ik}-b_{jk}\right)$$ (2)

so that $\sum_{i=1}^{n}\sum_{j=1}^{n}{\left(c_{ij}-b_{ij}\right)}^{2}$ is minimized, where the optimal transfer matrix C satisfies *C* = (*c*_*ij*_)_*n*×*n*_ (*i*, *j* = 1,2,3⋯*n*).

Step 4. Construct an optimization matrix $A^{*}={\left(a_{ij}^{*}\right)}_{n\times n}$, where $a_{ij}^{*}=10^{c_{ij}}$.

Step 5. Determine the subjective weight vector *ω*:

(1) Normalize the optimization matrix A* and get ${a_{ij}^{*}}^{\prime }$:

$${a_{ij}^{*}}^{\prime }=\frac{a_{ij}^{*}}{\sum_{i=1}^{n}a_{ij}^{*}}$$ (3)

(2) Add by row to get sum vector $\omega_{i}^{\prime }$:

$$\omega_{i}^{\prime }=\sum_{j=1}^{n}{a_{ij}^{*}}^{\prime }$$ (4)

(3) Normalize $\omega_{i}^{\prime }$ to obtain the characteristic vector of optimization matrix A*. That is the subjective weight vector *ω*_*i*_ obtained of the improved AHP:

$$\omega_{i}=\frac{\omega_{i}^{\prime }}{\sum_{i=1}^{n}\omega_{i}^{\prime }}$$ (5)

#### Improved TOPSIS

TOPSIS was first put forward by C.L.Hwang and K.Yoon in 1981. It is an evaluation method that ranks a limited number of evaluation objects according to their proximity to the ideal target [33]. TOPSIS is an effective multi-index evaluation method. It mainly focuses on selecting the optimal solution by constructing the optimal solution and the inferior solutions of the evaluation problem and calculating the relative sticking progress of each solution to the ideal solution [34]. However, due to the complexity of solving the canonical matrix and the subjectivity of attribute weights, it is necessary to improve this method. Since the purpose of this section is only to determine the objective weight, the relevant formulas related to decision analysis are not enrolled. The specific steps of the improved TOPSIS [35] are as follows:

Step 1. Standardization of decision matrix. Suppose there are *m* evaluation indexes and *n* index attribute values. Let *Y* = (*y*_*ij*_)_*m*×*n*_, *X* = (*x*_*ij*_)_*m*×*n*_,where *Y* is the decision matrix of multi-attribute decision-making problem, and *X* is the normalized decision matrix, and satisfies:

$$x_{ij}=\frac{y_{ij}}{\sqrt{\sum_{i=1}^{m}{y_{ij}}^{2}}},\;i=1,2,\cdots ,m;\;j=1,2,\cdots ,n$$ (6)

Step 2. Construct weighted gauge matrix *R* = (*r*_*ij*_)_*m*×*n*_. Let *ω*_*j*_ be the weight vector for each attribute, then

$$r_{ij}=\omega_{j}\times x_{ij}$$ (7)

Step 3. Construct the optimization model of goal programming.

$$min\;Z=\sum_{i=1}^{m}d_{i}^{+}=\sum_{i=1}^{m}\sum_{j=1}^{n}{\left(x_{ij}-x_{j}^{*}\right)}^{2}{\omega_{j}}^{2}$$

$$s.t.\{\begin{array}{c} \sum_{j=1}^{n}\omega_{j}=1\\ \omega_{j}\geq 0,\;j=1,2,\cdots ,n \end{array}$$ (8)

where $d_{i}^{+}$ is the distance from the alternative *d*_*i*_ to the positive ideal solution (optimal solution). $x_{j}^{*}$ satisfies: $x_{j}^{*}=\max\left\{x_{ij}1\leq i\leq m\right\}$, the index is the benefit-type index; $x_{j}^{*}=\min\left\{x_{ij}1\leq i\leq m\right\}$, the index is the cost-based index, the same is true for $r_{j}^{*}$ below.

Step 4. Make Lagrange function.


$$L(\omega ,\lambda )=\sum_{i=1}^{m}\sum_{j=1}^{n}{\left(r_{ij}-r_{j}^{*}\right)}^{2}+\lambda [\sum_{j=1}^{n}\omega_{j}-1]=\sum_{i=1}^{m}\sum_{j=1}^{n}{\left(x_{ij}-x_{j}^{*}\right)}^{2}{\omega_{j}}^{2}+\lambda [\sum_{j=1}^{n}\omega_{j}-1]$$ (9)

Let the partial derivative equal to 0, there is:

$$\{\begin{array}{c} 2\omega_{j}\sum_{i=1}^{m}{\left(x_{ij}-x_{j}^{*}\right)}^{2}-\lambda =0\\ \sum_{j=1}^{n}\omega_{j}-1=0 \end{array}$$ (10)

Finally, *ω*_*j*_ is solved.


$$\omega_{j}={\left\{[\sum_{j=1}^{n}\frac{1}{\sum_{i=1}^{m}{\left(x_{ij}-x_{j}^{*}\right)}^{2}}]∙\sum_{i=1}^{m}{\left(x_{ij}-x_{j}^{*}\right)}^{2}\right\}}^{-1}$$ (11)

### Combination weight of game theory

After solving the subjective and objective weights respectively, the two weights are combined with the idea of Game Theory. At present, the commonly used synthesis methods are additive synthesis, multiplicative synthesis, etc. This article selects the target of minimalization of the deviation, combines the obtained two weights, and finally get the combination weight value [36]. The specific steps are as follows:

(1) When two methods are used to calculate the weights of evaluation indexes, the weights need to be combined. In order not to lose the generality of the method, this paper uses the parameter *L* to denote the type of weighting method. Thus the weight vector can be expressed as $\omega_{k}=(\omega_{k1},\omega_{k2},\cdots ,\omega_{kl})$. Since only two methods are used in this paper to calculate the weights of evaluation indexes, the following *L* = 2. The linear combination of *L*′*s* own vectors is $\omega =\sum_{k=1}^{L}\alpha_{k}{\omega_{k}}^{T}$. In order to get the optimal combination weight ω*, it is necessary to optimize the linear combination. Therefore, the game model is introduced as follows:

$$\min\left\|\sum_{k=1}^{L}\alpha_{k}{\omega_{k}}^{T}-\omega_{k}\right\|$$ (12)

(2) According to the differential property of matrix, the linear Eqs of optimal derivative conditions are as follows:

$$\left[\begin{array}{c} \omega_{1}{\omega_{1}}^{T}\;\cdots \;\omega_{1}{\omega_{L}}^{T}\\ \vdots \;\vdots \\ \omega_{L}{\omega_{1}}^{T}\;\cdots \;\omega_{L}{\omega_{L}}^{T} \end{array}][\begin{array}{c} \alpha_{1}\\ \vdots \\ \alpha_{L} \end{array}]=[\begin{array}{c} \omega_{1}{\omega_{1}}^{T}\\ \vdots \\ \omega_{L}{\omega_{L}}^{T} \end{array}\right]$$ (13)

(3) The obtained coefficients *α* are normalized to obtain α*, and finally the combination weight ω* is:

$$\omega^{*}=\sum_{k=1}^{L}{\alpha_{k}}^{*}{\omega_{k}}^{*}$$ (14)

### Comprehensive evaluation model based on Game Theory-cloud model

Intelligent cockpit evaluation is not only a multi-attribute and multi-standard decision-making problem, but also fuzzy and random among evaluation indexes [18]. Although a reasonable weight value can be obtained from the above theory, there are many limitations and deficiencies in evaluating comfort level only by means of weighting. In order to make the transformation between qualitative and quantitative easier and realize interchangeability [37], the cloud model is introduced in this paper.

The word "cloud model" first appeared in 1995 and was put forward by Academician Li Deyi of China Academy of Sciences [38]. Cloud model is a new hybrid model, which is a more complete and effective cognitive technology compared to the affiliation function. The cloud model is developed and formed on fuzzy mathematics and probability theory. It fully considers the data characteristics of the evaluation object and better solves the fuzziness and randomness in the self-evaluation problem. The current application scenarios of cloud model are very extensive: artificial intelligence [39], evaluation and analysis [18, 40], data mining [41].

#### Basic theory of cloud model

**Definition 1** For an ordinary set *U*, *U* is said to be a discourse of universe if it satisfies *U* = {*x*} [38, 39]. Let *U* be a quantitative discourse of universe expressed in numerical values and *C* be a qualitative concept on *U*. If a quantitative value *x*∈*U* is a random realization of a qualitative concept *C*, *x* is a random number with stable tendency for *C* with determinacy *μ*(*x*)∈[0,1]. It is mathematically expressed as μ:U→[0,1],∀x∈U,x→μ(x). Then the distribution of *x* over the discourse of universe *U* is called a cloud, denoted as cloud *C*(*X*). Each *x* is called a cloud droplet. A cloud is composed of a number of cloud droplets. They are a single random realization of a qualitative concept, and multiple generated cloud droplets can synthesize the overall characteristics of this qualitative concept [19].

**Definition 2** The cloud model describes the properties of clouds by defining three numerical features *C*(*Ex*、*En*、*He*). Among them, expectation *Ex* represents the expectation value of cloud droplets in the spatial distribution of the universe of discourse. It is the center point on the universe of discourse and represents the overall characteristics of a qualitative concept. Entropy *En* represents the fuzziness measure of the qualitative concepts. It is used to describe the span of the clouds, and reflect the dispersion of cloud droplets. Hyper entropy *He* represents the uncertainty measure of entropy, which is used to describe the uncertainty degree of cloud droplets.

**Definition 3** If the random variable *x* satisfies: *x*~*N* (*Ex*, *En*′^2^), where *En*′~*N* (*En*, *He*^2^)and *En*≠0, there is:

$$y=e^{-\frac{{\left(x-Ex\right)}^{2}}{2{En}^{2}}}$$ (15)

where *y* is referred to as the expectation curve of a cloud model.

According to the definition of expectation curve, after the three cloud parameters are determined, the corresponding cloud diagram can be drawn uniquely. Referring to the division of interval evaluation grades in Table 1, the domain of index evaluation is divided into five sub-intervals. The parameters corresponding to each interval are:

$$\{\begin{array}{c} Ex=\frac{d_{min}+d_{max}}{2}\\ En=\frac{d_{max}-d_{min}}{6}\\ He=\beta \end{array}$$ (16)

among them, *d*_*min*_ and *d*_*max*_ represent the end points of the scoring interval according to the comfort evaluation level. That is, each level interval can be represented as [*d*_*min*_, *d*_*max*_]. *β* as random numbers, this article takes *β* = 0.05.

#### Calculation of evaluation cloud parameters

The interval values of five grades are respectively brought into Eq (16) to obtain a set of cloud parameters for the standard evaluation cloud. The calculation results are shown in Table 2.

**Table 2:** Standard evaluation cloud parameters.

Grade	Score interval	*Ex*	*En*	*He*
**Ⅰ**	[0,2]	1	0.33	0.05
**Ⅱ**	[2,4]	3	0.33	0.05
**Ⅲ**	[4,6]	5	0.33	0.05
**Ⅳ**	[6,8]	7	0.33	0.05
**Ⅴ**	[8,10]	9	0.33	0.05

The first column indicates the comfort level. The second column indicates the score interval corresponding to the comfort level. The rightmost three columns contain the three parameters corresponding to different standard clouds.

Once the three parameters of the standard evaluation cloud have been determined, it is possible to qualitatively compare the closeness of each second-class index relative to the standard evaluation cloud. However, in order to obtain the comprehensive comfort of the cockpit, it is necessary to calculate the comprehensive cloud. Set the evaluation index cloud of the second-class index to get the matrix $Z_{i}=(z_{i1},z_{i2},\cdots ,z_{im},i=1,2,\cdots ,n)$ by expert scoring. Where *m* is the number of indexes and *n* is the number of experts. Assuming that the *j*-th evaluation index cloud of the second-class index is *C*_*j*_(*Ex*_*j*_, *En*_*j*_, *He*_*j*_), there is:

$$\{\begin{array}{c} {Ex}_{j}=\frac{1}{n}\sum_{i=1}^{n}z_{ij}\\ {En}_{j}=\frac{1}{n}\sqrt{\frac{\pi }{2}}\sum_{i=1}^{n}\left|z_{ij}-{Ex}_{j}\right|\\ S_{j}^{2}=\frac{1}{n-1}\sum_{i=1}^{n}{\left(z_{ij}-{Ex}_{j}\right)}^{2}\\ {He}_{j}=\sqrt{\left|S_{j}^{2}-{{En}_{j}}^{2}\right|} \end{array}$$ (17)

where $S_{j}^{2}$ represents the variance of the expert score of the *j*-th index.

Due to the difference of experts’ cognition, a floating cloud algorithm is used to address the conceptual differences in reference [42]. The combination weight solved by the Game Theory are used to obtain the cloud parameters of the first-class indexes:

$$\{\begin{array}{c} Ex=\frac{{Ex}_{1}\omega_{1}+{Ex}_{2}\omega_{2}+\cdots +{Ex}_{n}\omega_{n}}{\sum_{i=1}^{n}\omega_{i}}\\ En=\frac{{En}_{1}{\omega_{1}}^{2}+{En}_{2}{\omega_{2}}^{2}+\cdots {En}_{n}{\omega_{n}}^{2}}{\sum_{i=1}^{n}{\omega_{i}}^{2}}\\ He=\frac{{He}_{1}{\omega_{1}}^{2}+{He}_{2}{\omega_{2}}^{2}+\cdots {He}_{n}{\omega_{n}}^{2}}{\sum_{i=1}^{n}{\omega_{i}}^{2}} \end{array}$$ (18)

#### Cloud model similarity measurement and optimization

According to Eq (18), the evaluation index cloud parameters corresponding to the first-class index can be calculated. In order to get the relationship between the comprehensive environment and cockpit comfort, the cloud parameters of the comprehensive evaluation cloud also need to be calculated. By qualitatively analyzing the intersection of different evaluation clouds, a rough evaluation result can be derived. However, in order to quantify the degree of intersection between cloud models, the similarity measurement method is utilized to quantitatively analyze the evaluation results. In this paper, ECM and MCM are selected to calculate the similarity, and a more optimized calculation method is proposed by combining the above two algorithms.

Referring to the similarity calculation method in reference [19], assuming that two cloud models are *C*_1_(*Ex*_1_, *En*_1_, *He*_1_) and *C*_2_(*Ex*_2_, *En*_2_, *He*_2_) respectively. Then the calculation formula based on the similarity between the expectation curve and the maximum boundary curve is defined as:

$$\{\begin{array}{c} ECM(C_{1},C_{2})=\frac{2S}{\sqrt{2\pi }({En}_{1}+{En}_{2})}\\ MCM(C_{1},C_{2})=\frac{2S}{\sqrt{2\pi }({en}_{1}+{en}_{2})} \end{array}$$ (19)

in the above Eq, *en*_*i*_ = 3*He*_*i*_+*En*_*i*_(*i* = 1,2), which is determined by the expected curve based on the maximum boundary method. The letter *S* denotes the area of the intersection of two clouds. The derivation process of the specific two intersecting clouds’ area calculation method is referred to the reference [19].

The degree of similarity between the two cloud models can be quantified separately according to Eq (19). But ECM does not introduce *He* for calculation, which leads to some errors between the calculated results and theoretical values. While MCM, though introducing 3*He* for calculation, it overemphasizes the influence of *He*. Therefore, this paper defines a similarity measure method of Expectation curve and Maximum boundary curve based on Cloud Model (EMCM) based on the two theories:

$$EMCM(C_{1},C_{2})=\alpha ECM+\beta MCM$$ (20)

where *α* and *β* are similarity weights based on the expectation curve and the maximum boundary curve, respectively. *α* and *β* are determined as follows:

$$\{\begin{array}{c} d_{1}=\sqrt{{\left(ECM-MCM\right)}^{2}}\\ d_{2}={\left(\alpha -\beta \right)}^{2}\\ d_{1}=d_{2}\\ \alpha +\beta =1\\ \alpha \geq \beta \end{array}$$ (21)

where *d*_1_、*d*_2_ is the distance function introduced to study the relationship between the two weights. In order to make *ECM*、*MCM* with *EMCM* the degree of difference is consistent with the difference of the corresponding distribution coefficients, this paper takes *d*_1_ = *d*_2_.

By calculating the similarity values between the index evaluation clouds and the five standard evaluation clouds, the evaluation results for a certain working condition of the research object are derived.

## Application example and simulation analysis of evaluation model of automobile intelligent cockpit

**Fig 2:**
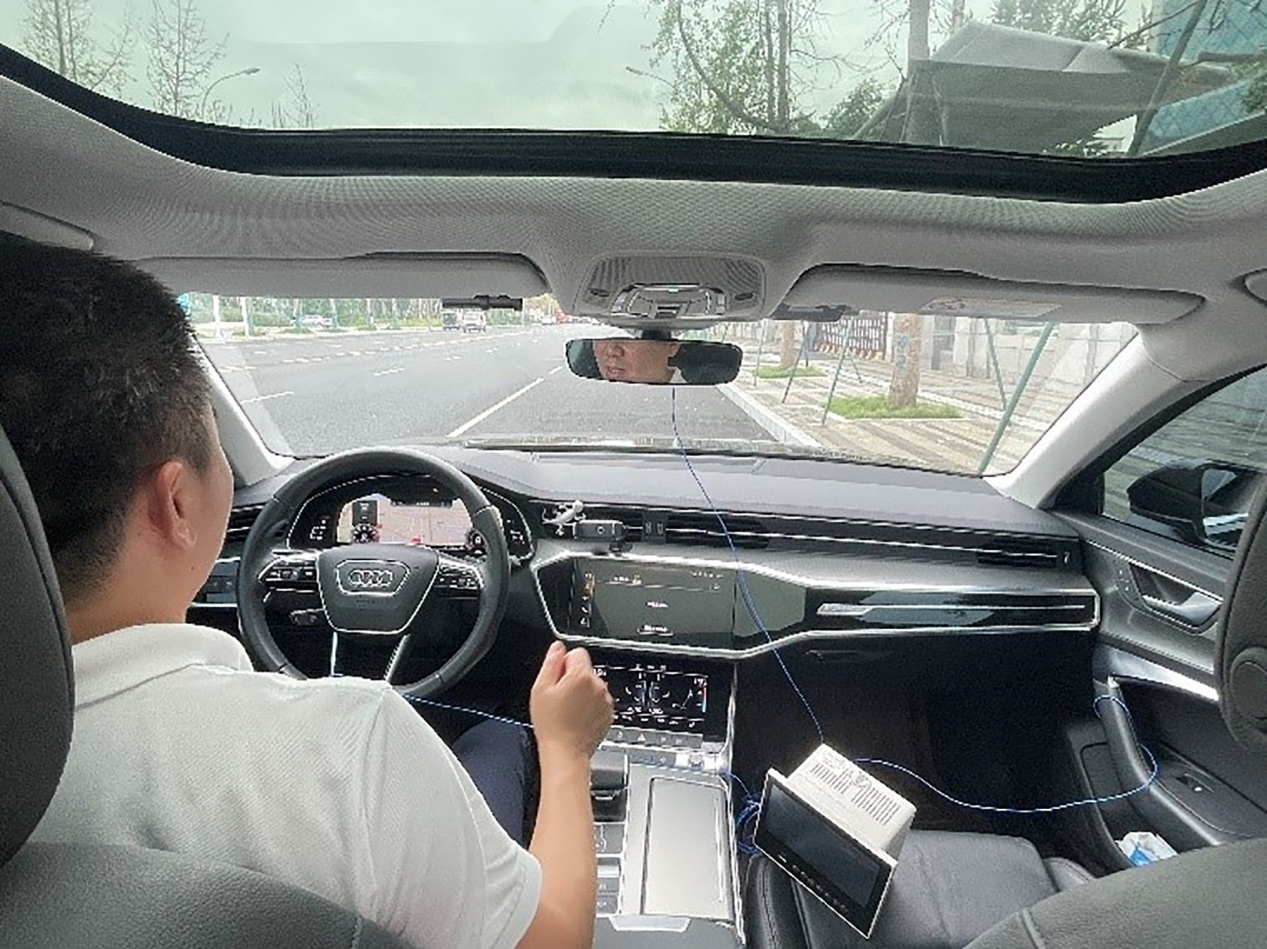
Example diagram of Audi’s working condition. This figure shows the interior of a 2021 Audi A6. The vehicle is used to verify the correctness and reasonableness of the improved combination weighting-cloud model developed based on this paper.

To evaluate whether the model is reasonable, an engineering example is needed for auxiliary verification. In this paper, a 2021 Audi A6 vehicle is selected based on the consideration of comprehensive factors, as shown in Fig 2. The working conditions of this car are known as: the vehicle speed 38.7–42.1 km/h, the cockpit temperature 22.6–24.5°C, and the vehicle price is 418,000 yuan [43, 44]. Showed that the car was in a uniform speed working condition for most of the driving time. Studying the uniform speed working condition can provide a more stable testing environment for participants and reduce the influence of other factors [45]. In this paper, we refer to the general automobile comfort test method to study the comfort level of the cockpit of a car under uniform driving conditions. A total of 8 experimenters were selected to participate in the experiment. Each experimenter is an expert in the automobile industry. Each expert scored each index of cockpit comfort separately, and the scoring value was specified in the interval [0,10].

### Calculation of weights

**Fig 3:**
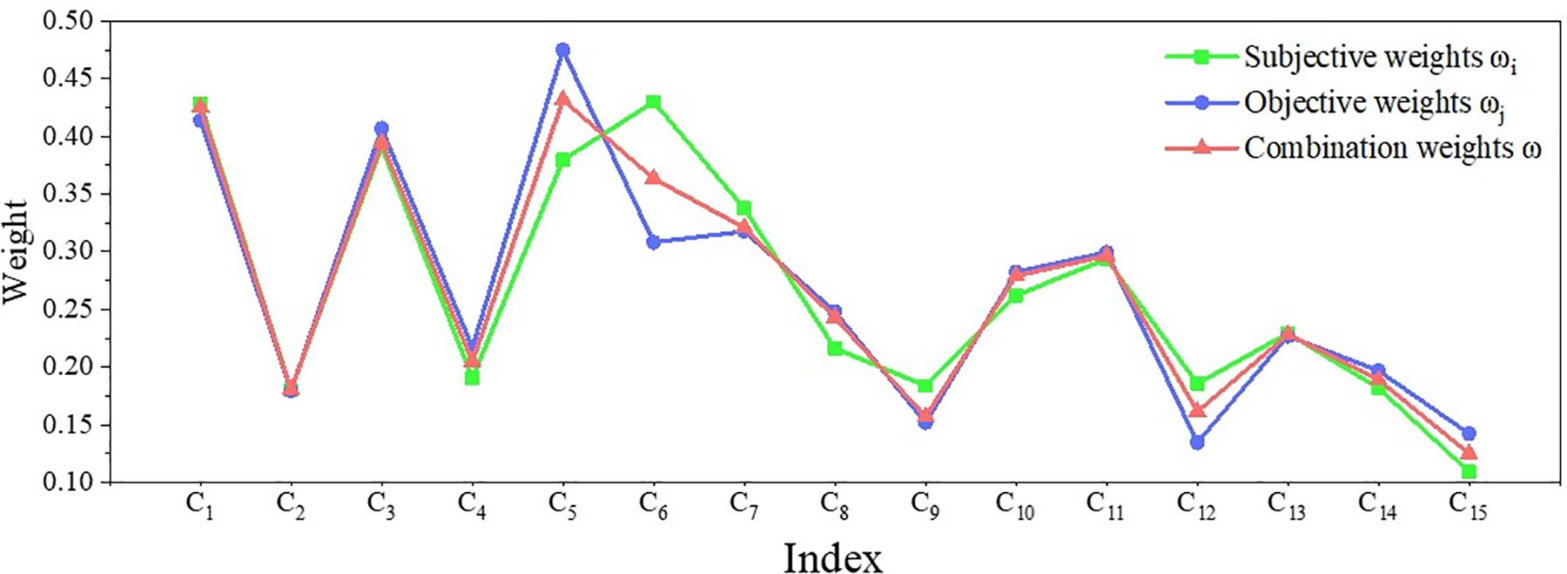
Index weight reference comparison chart. The data in Table 3 are presented in the form of line graphs. There are three curves in the graph, where green represents the subjective weights, blue represents the objective weights, and red represents the combination weights.

According to the evaluation system of automobile intelligent cockpit comfort established above, a weight questionnaire of each index of automobile cockpit comfort was firstly made. A total of 237 people participated in the questionnaire, all of whom were automotive professionals in the school, including students and teachers. The number of invalid tickets is 8, the number of valid tickets is 229, and the effective rate is 96.62%. Participants need to compare and score the importance of each second-class index, so as to determine the relative importance of each second-class index. The obtained judgment matrix *A* obtained from S1–S4 Tables is brought into Eqs (1–5), and the subjective weight is obtained according to the improved AHP. Eight experts were invited to score the cockpit comfort environment corresponding to each second-class index environment, and the decision matrix *Y* was obtained. Bring the data into Eqs (6–11), and get the objective weight according to the improved TOPSIS. Through the idea of Game Theory, the subjective weight and objective weights are combined to get the final combination weights. The weight data is shown in Table 3. In order to visually compare the relationship among the three weights, draw a weight reference comparison chart, as shown in Fig 3. As can be seen from Fig 3, the combination weights are between subjective weights and objective weights. It considers the actual requirements of subjective and objective respectively, which is a group of more optimized weights.

**Table 3:** Index weights and index cloud parameters.

Index	Subjective weights	Objective weights	Combination weights	Index cloud parameters
***C***_**1**_	0.4285	0.4137	0.4254	(8.51,0.30,0.28)
***C***_**2**_	0.1804	0.1795	0.1802	(7.79,0.42,0.37)
***C***_**3**_	0.3911	0.4068	0.3944	(8.23,0.34,0.32)
***C***_**4**_	0.1906	0.2169	0.2050	(7.53,0.47,0.41)
***C***_**5**_	0.3795	0.4748	0.4317	(8.76,0.30,0.28)
***C***_**6**_	0.4299	0.3083	0.3634	(8.45,0.25,0.24)
***C***_**7**_	0.3376	0.3176	0.3209	(8.81,0.36,0.31)
***C***_**8**_	0.2162	0.2481	0.2429	(8.35,0.41,0.38)
***C***_**9**_	0.1840	0.1519	0.1572	(8.13,0.36,0.33)
***C***_**10**_	0.2622	0.2824	0.2791	(8.54,0.31,0.29)
***C***_**11**_	0.2939	0.2994	0.2965	(8.58,0.41,0.36)
***C***_**12**_	0.1856	0.1345	0.1615	(7.63,0.38,0.35)
***C***_**13**_	0.2294	0.2269	0.2282	(8.53,0.32,0.29)
***C***_**14**_	0.1819	0.1968	0.1889	(8.68,0.41,0.38)
***C***_**15**_	0.1093	0.1423	0.1249	(7.60,0.41,0.38)

The first four columns in the table include subjective weights, objective weights and combination weights based on Game Theory. And the last one column of the table lists the three parameters of the cloud model for each second-class index to calculate the similarity value with the standard cloud.

### Standard evaluation cloud

**Fig 4:**
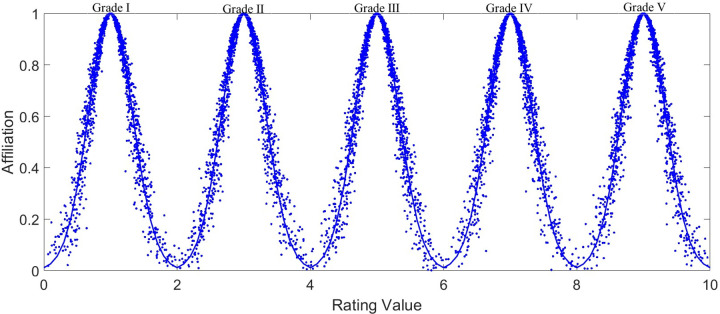
Standard index cloud. The parameters of five standard clouds are respectively input into the program of forward cloud model. Input the total number of 1500 particles, set the expectation curve to blue, and draw the cloud diagram corresponding to the standard cloud.

According to the principle of forward cloud model, the standard evaluation cloud parameters obtained in Table 2 are used as input. Determine the total number of cloud droplets *N* = 1,500, and draw the standard evaluation cloud diagram of cockpit comfort evaluation, as shown in Fig 4. From left to right in the figure, the comfort level of cockpit is GradeⅠ-Ⅴ. The specific descriptions of each grade are shown in Table 2.

### Index evaluation cloud

Eight experts evaluated and scored the second-class indexes under different working conditions. The specific scoring results are shown in Table 4.

**Table 4:** Second-class index scoring.

Index		Expert 1	Expert 2	Expert 3	Expert 4	Expert 5	Expert 6	Expert 7	Expert 8
***B***_**1**_	***C***_**1**_	9.2	8.5	8.6	8.2	8.4	8.3	8.7	8.2
	***C***_**2**_	8.7	7.5	8.1	7.9	7.4	7.6	7.8	7.3
	***C***_**3**_	8.5	8.1	8.3	7.6	8.8	8.4	7.9	8.2
***B***_**2**_	***C***_**4**_	7.0	7.6	7.9	8.4	7.1	7.3	7.2	7.7
	***C***_**5**_	9.2	8.3	8.9	8.7	9.1	8.8	8.5	8.6
	***C***_**6**_	8.8	8.2	8.5	8.3	8.7	8.6	8.2	8.3
***B***_**3**_	***C***_**7**_	8.9	8.8	9.5	9.1	8.1	8.4	8.8	8.9
	***C***_**8**_	7.8	8.1	8.8	8.5	7.9	8.8	8.3	8.6
	***C***_**9**_	8.2	8.7	8.3	7.8	7.6	8.5	7.9	8.1
	***C***_**10**_	8.5	8.4	9.1	8.8	8.0	8.3	8.7	8.5
***B***_**4**_	***C***_**11**_	8.0	8.1	8.5	8.8	9.3	8.6	8.4	8.9
	***C***_**12**_	7.6	8.4	7.5	7.1	7.3	7.8	7.4	7.9
	***C***_**13**_	8.5	9.3	8.7	8.1	8.5	8.2	8.6	8.3
	***C***_**14**_	8.2	9.1	8.3	8.3	9.2	8.9	8.8	8.6
	***C***_**15**_	6.9	7.3	7.9	7.8	7.5	8.1	7.9	7.4

The table shows the level of comfort of experts for each second-class index of the automobile cockpit under specific working conditions. A total of eight experts were involved in the scoring, four of them were men and four were women.

The data in Table 4 are substituted into the Eq (17) to obtain the second-class index cloud parameter *C*_*j*_(*Ex*_*j*_, *En*_*j*_, *He*_*j*_), as shown in Table 3. The cloud parameters of the second-class index cloud calculated in Table 3 and the corresponding weights are brought into Eq (18) to obtain the cloud parameters of the first-class index cloud. The same method is used to determine the weights of the first-class indexes. The level of importance between two levels of indexes has been organized in a table, which is stored in Supporting Information. The first-class index determined by the experiment (*B*_1_, *B*_2_, *B*_3_, *B*_4_) with the first-class weights is (0.2496,0.1796,0.2585,0.3123). The index cloud parameters and corresponding weights of the first-class index are brought into the Eq (18) to find the cloud parameters of the comprehensive evaluation cloud. It is worth noting that the generation of comprehensive evaluation cloud does not rely on the role of a single variable. And the establishment process of its three parameters is jointly determined by all variables.

**Fig 5:**
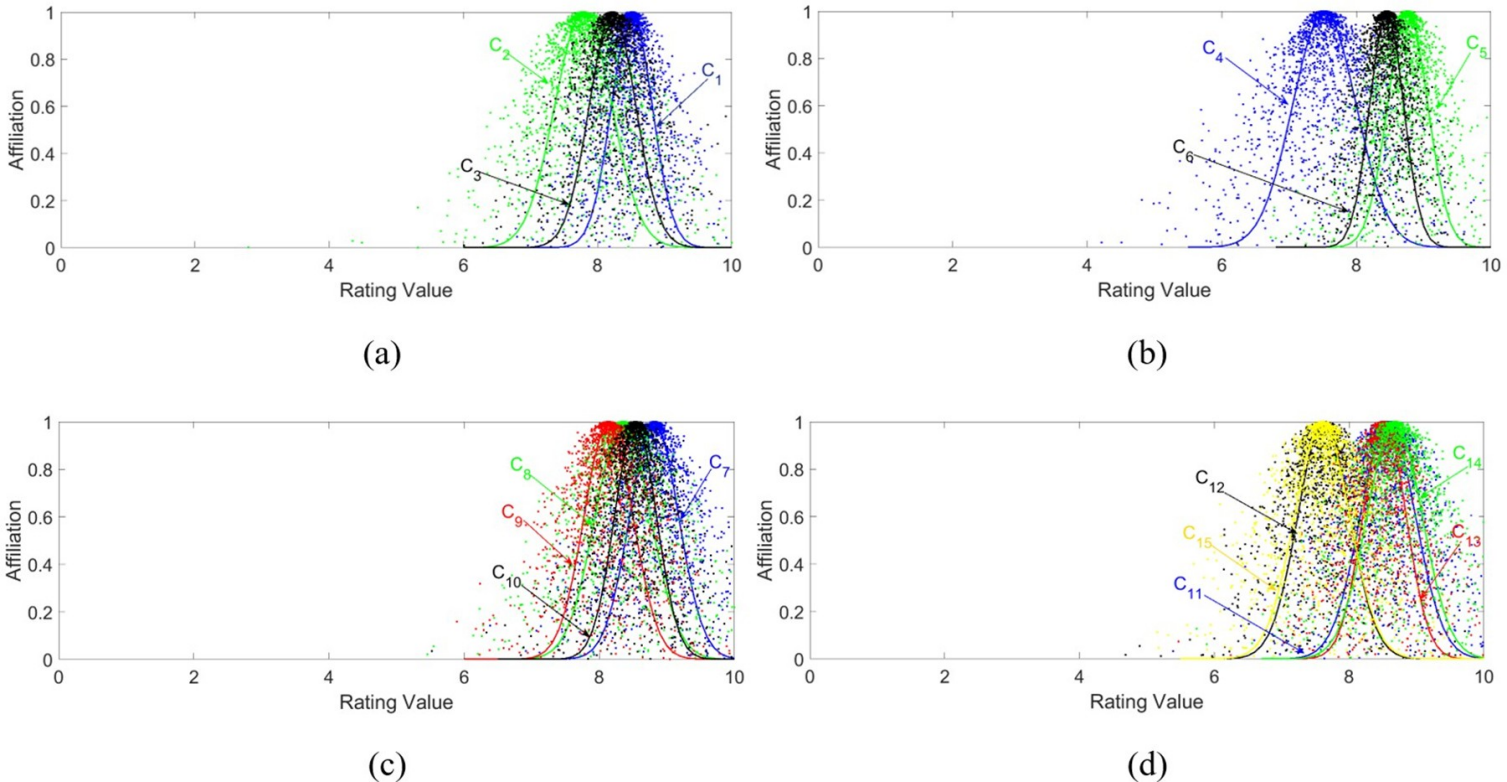
Cloud diagram of second-class indexes. The indexes are placed in cloud diagrams (a), (b), (c) and (d) according to the major categories. (a), (b), (c) and (d) indicate the clouds of noise and vibration, light environment, thermal environment, and human-computer interaction second-class indexes, respectively.

**Fig 6:**
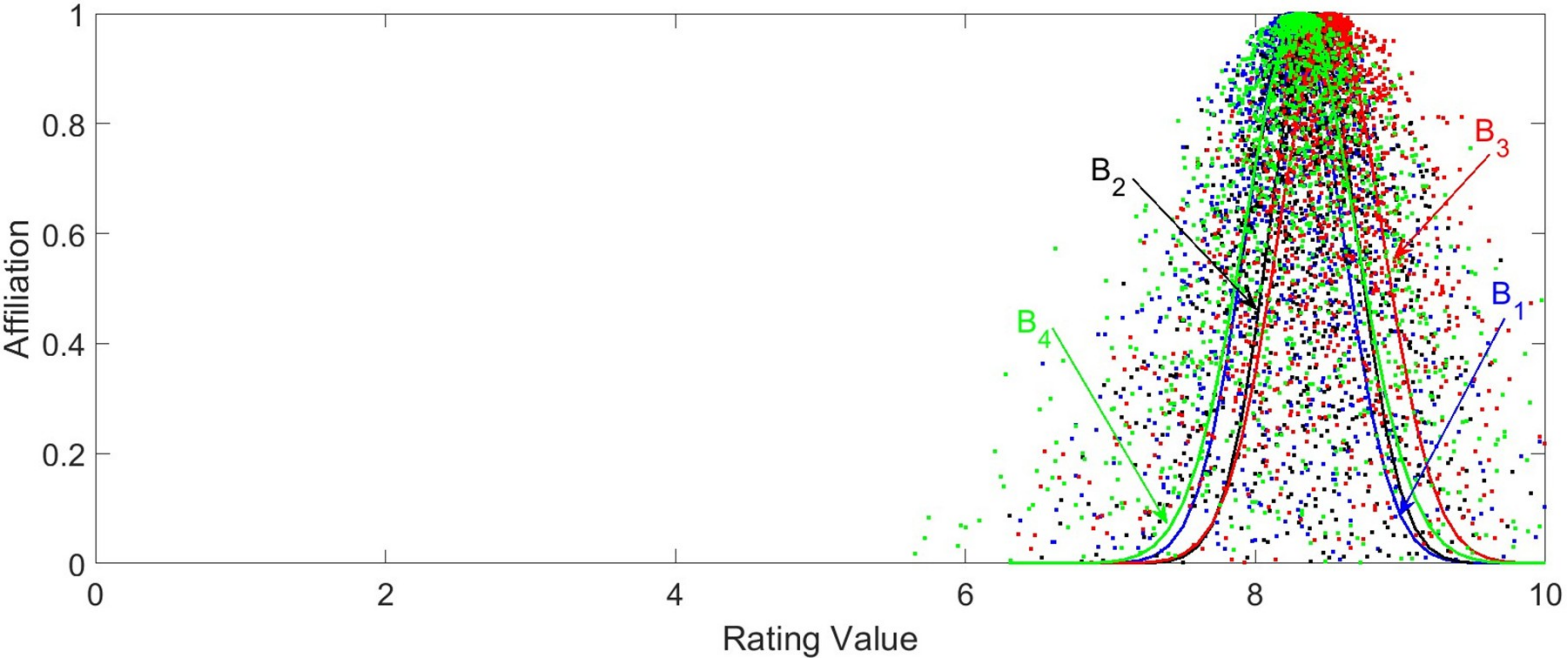
Cloud diagram of first-class indexes. The blue color indicates the cloud diagram of noise and vibration. The black color indicates the cloud diagram of light environment. The red color indicates the cloud diagram of thermal environment. And the green color indicates the cloud diagram of human-computer interaction.

Based on the cloud model parameters in Table 3, the cloud diagrams of first-class and second-class indexes can be drawn by using Eq (15), as shown in Figs 5 and 6.

The comfort grade corresponding to each index can be observed very visually according to the cloud diagram. From Fig 5, it is easy to compare that *C*_2_ in (a), *C*_4_ in (b), *C*_12_ and *C*_15_ in (d) are satisfied 6>*Ex*<8. It means that the overall situation corresponding to the four indexes is in the interval value of (6, 8], which is slightly uncomfortable. Through the cloud diagram, it can be intuitively analyzed that under the working conditions of the vehicle, the thermal environment is in a relatively comfortable situation. The expectation values of the thermal environment corresponding to the second-class indexes are all in the range of (8,10]. Thus, it can be inferred that this type of vehicle has a high ability to regulate the thermal environment. Similarly, the cloud diagram shown in Fig 6 can also be used to analyze the state of the vehicle cockpit. It is not difficult to compare $Ex(B_{3})>Ex(B_{2})>Ex(B_{4})>Ex(B_{1})>8$. So it is judged that the cockpit of the vehicle is in a comfortable state, and the thermal environment inside the vehicle is the best.

**Fig 7:**
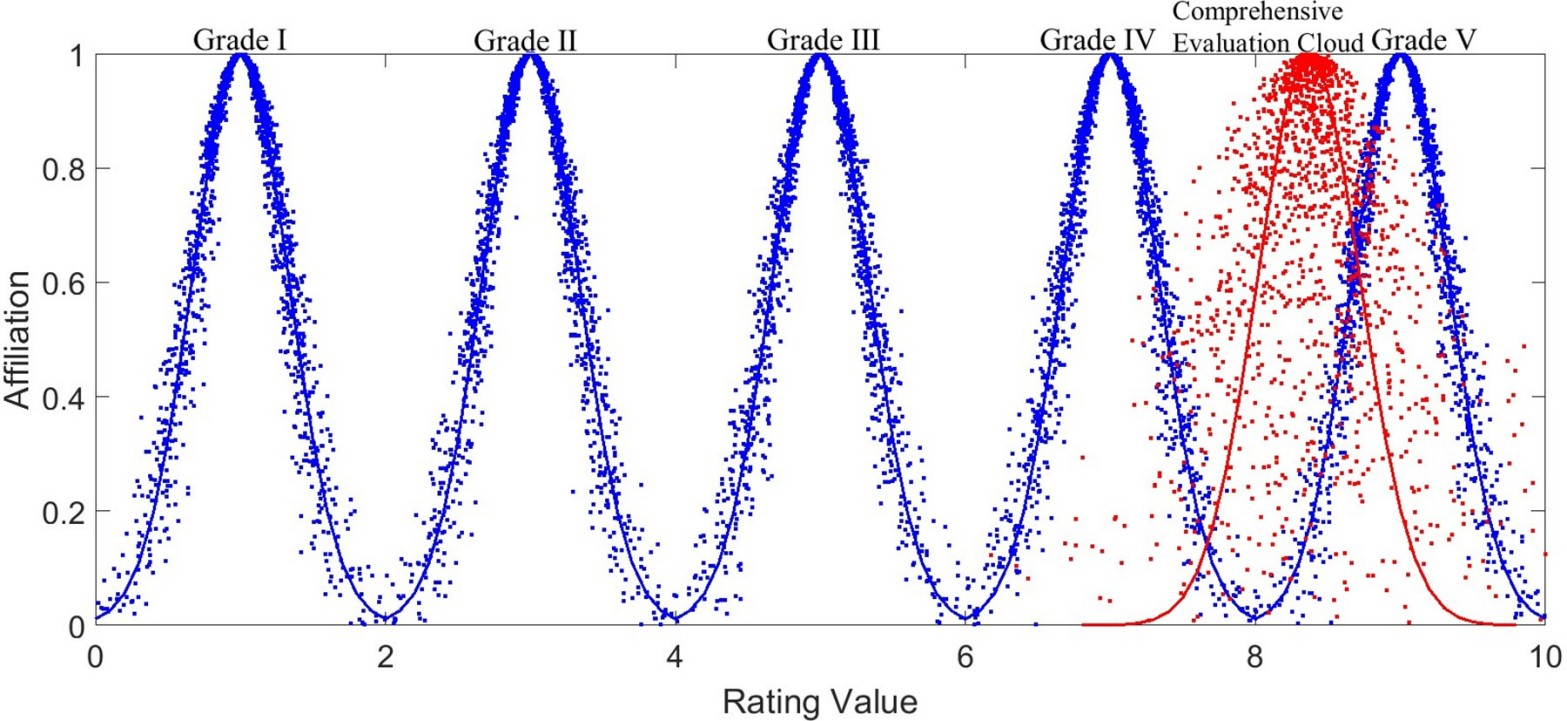
Comprehensive evaluation cloud. The figure shows the condition of the intersection of the composite cloud and the 5 standard clouds. The red color represents the comprehensive evaluation cloud and the blue color represents the index standard cloud.

The comprehensive evaluation cloud is defined as *C*_*Z*_. Based on the idea of floating cloud, the comprehensive evaluation cloud *C*_*Z*_(8.3677,0.3534,0.3226) is finally calculated. The comprehensive evaluation cloud and the standard index cloud can be drawn in the same cloud diagram, as shown in Fig 7.

From Fig 7, its comprehensive evaluation cloud has the largest intersection area with the Ⅴ-grade cloud. This indicates that the comprehensive evaluation cloud has the highest similarity with this cloud, so the comprehensive evaluation of this automobile cockpit is comfortable.

### Cloud similarity and evaluation level

The above steps can only roughly determine or qualitatively analyze whether the cockpit is comfortable, but can’t truly quantify the comfort value. The parameters of comprehensive evaluation cloud and standard index cloud are substituted into Eq (19). So, the similarity value based on ECM and MCM algorithm is calculated.

Then use Eq (21) to determine the values of the parameters *α*、*β*, as shown in Table 5. After the parameters are determined, they are brought into Eq (20) to calculate the similarity value obtained by EMCM method, as shown in Table 6.

**Table 5:** Two parameters of EMCM.

Grade	Ⅰ	Ⅱ	Ⅲ	Ⅳ	Ⅴ
**0303*α***	0.5000	0.5000	0.6219	0.7974	0.6989
***β***	0.5000	0.5000	0.3781	0.2026	0.3011

Two parameters of EMCM are shown in Table 5. Parameters correspond to the relative weights of ECM and MCM respectively.

**Table 6:** Three similarity calculation methods calculate values.

Grade	Ⅰ	Ⅱ	Ⅲ	Ⅳ	Ⅴ
**ECM**	0	0	0	0.0429	0.3592
**MCM**	0	0	0.0594	0.3968	0.5175
**EMCM**	0	0	0.0225	0.1146	0.4069

Table 6 shows the similarity values of the standard evaluation cloud and the index evaluation cloud calculated based on the three methods.

The magnitude of the similarity value characterizes the correlation and the progress of the cloud to cloud. The larger the similarity value indicates the higher correlation between two clouds, and vice versa, the lower the correlation. From Table 6, the similarity value between the comprehensive evaluation cloud and the Ⅴ-grade standard evaluation cloud calculated based on the three methods is the largest. This is Followed by the Ⅳ-grade standard evaluation cloud and the Ⅲ-grade standard evaluation cloud. And the similarity between the remaining grades and the comprehensive evaluation cloud is 0. From the quantitative point of view, the automobile cockpit can be evaluated as comfortable.

Analyzing the data in Table 6, the similarity values of its standard evaluation cloud and index evaluation cloud are: ECM (Ⅴ>Ⅳ>Ⅲ>Ⅱ>Ⅰ), MCM (Ⅴ>Ⅳ>Ⅲ>Ⅱ>Ⅰ), and EMCM (Ⅴ>Ⅳ>Ⅲ>Ⅱ>Ⅰ). That is, the direction of the results obtained using the three similarity calculation methods is the same. And the similarity value is the most similar to the standard evaluation cloud of Ⅴ-grade. From the definitions in Table 1, the automobile cockpit can be evaluated as comfortable. To further verify its accuracy, a fuzzy algorithm was used to verify its comparison.

### Verification and evaluation of the model

Considering the multi-attribute and fuzziness of comfort index of intelligent cockpit, this paper selects multi-level fuzzy comprehensive evaluation method to verify the correctness and rationality of the model. The specific steps are as follows:

Step 1. Construct the membership matrix of 15 second-class indexes.


$$R={\left[\begin{array}{c} 0.90\;0.10\;0.00\;0.00\;0.00\\ \cdots \;\cdots \;\cdots \;\cdots \;\cdots \\ 0.70\;0.15\;0.05\;0.05\;0.05\\ \cdots \;\cdots \;\cdots \;\cdots \;\cdots \\ 0.70\;0.10\;0.05\;0.05\;0.05 \end{array}\right]}_{15\times 5}$$

Step 2. Combine the second-class weights to perform a first-class fuzzy comprehensive evaluation on the sub-index set:

$$B_{1}=[0.8820\;0.0713\;0.0287\;0.0090\;0.0090]$$

$$B_{2}=[0.8250\;0.0784\;0.0500\;0.0284\;0.0182]$$

$$B_{3}=[0.7478\;0.1043\;0.0640\;0.0500\;0.0340]$$

$$B_{4}=[0.7855\;0.1000\;0.0562\;0.1018\;0.1018]$$

Step 3. Combine the first-class weights to perform a second-class fuzzy comprehensive evaluation on the sub-index set:

$$B=[0.2496\;0.1796\;0.2585\;0.3123]∙\left[\begin{array}{c} \begin{array}{c} 0.8820\;0.0713\;0.0287\;0.0090\;0.0090\\ 0.8250\;0.0784\;0.0500\;0.0284\;0.0182\\ 0.7478\;0.1043\;0.0640\;0.0500\;0.0340 \end{array}\\ 0.7855\;0.1000\;0.0562\;0.1018\;0.1018 \end{array}\right]=[0.8069\;0.0901\;0.0502\;0.0521\;0.0461]$$

According to the principle of maximum affiliation, the maximum value is 0.8069. It corresponds to the comfort grade Ⅴ. So the evaluation of the intelligent cockpit of the automobile is comfortable. Based on this, this paper verifies the correctness of the intelligent cockpit comfort evaluation model based on the improved combination weighting-cloud model. Comparing the two methods, firstly, the fuzzy judgment matrix established by the fuzzy comprehensive evaluation method will change with the change of affiliation degree matrix. Secondly, the selection of the affiliation degree is generally a direct subjective assignment or an empirical formula. So, fuzzy comprehensive evaluation method can’t reflect the actual situation very well. In this paper, the affiliation degree is transformed into three parameters of cloud model, which reflects the actual situation more comprehensively. Therefore, the evaluation model based on the cloud model is more general.

## Conclusion and discussion

In this paper, the comfort evaluation system of intelligent cockpit is established according to the objective reality. The evaluation model of automobile intelligent cockpit based on improved combination weighting-cloud model is constructed. A more optimized similarity calculation method is defined in combination with the area-based similarity solving method. And the accuracy of the model is verified by engineering examples and fuzzy evaluation. The results show that:

1. The subjective and objective weights obtained based on the improved combination weighting method can make up for the limitation of single weighting, and it is a more ideal weighting method.
2. Compared with fuzzy evaluation in a single field, the evaluation model based on combination weighting-cloud model is more referential and can better reflect the fuzziness and randomness existing in the evaluation system.
3. Compared with using ECM or MCM alone, EMCM algorithm based on ECM and MCM has certain advantages. It makes up for the deficiency that ECM does not consider hyper-entropy when solving local features, and eliminates the influence of MCM’s over-emphasis on hyper-entropy. This method is more reasonable.

But at the same time, there are still many limitations in this paper:

1. In this paper, only 4 first-class indexes and 15 second-class indexes are selected for research. The research on the comfort system of automobile intelligent cockpit can be extended to the indexes of smell, vehicle type and air quality.
2. The number of working conditions and examples selected in this paper is too small, so different types of intelligent vehicles can be considered for research. In this paper, the working condition is also studied in a steady state, and the non-steady state or extreme situation can be considered to test the cockpit comfort of the vehicle.
3. Due to space reasons, this paper does not specifically analyze the affiliation between the second-class index cloud and the standard index cloud. Readers can draw the cloud diagrams of a single second-class index cloud and standard index cloud. Finally, the comfort status of a single index can be analyzed qualitatively and quantitatively through the cloud diagram and the optimized similarity algorithm established in this paper.

## Supporting information

S1 Table The judgment matrix data of noise and vibration. The table contains the judgment matrix of the second-class indexes *C*_1_−*C*_3_. It is used to obtain the corresponding second-class weights.
(DOCX)

S2 Table The judgment matrix data of light environment. The table contains the judgment matrix of the second-class indexes *C*_4_−*C*_6_. It is used to obtain the corresponding second-class weights.
(DOCX)

S3 Table The judgment matrix data of thermal environment. The table contains the judgment matrix of the second-class indexes *C*_7_−*C*_10_. It is used to obtain the corresponding second-class weights.
(DOCX)

S4 Table The judgment matrix data of human-computer interaction. The table contains the judgment matrix of the second-class indexes *C*_11_−*C*_15_. It is used to obtain the corresponding second-class weights.
(DOCX)

S5 Table The judgment matrix data of first-class index. The table contains the judgment matrix of the first-class indexes *B*_1_−*B*_4_. It is used to obtain the corresponding first-class weights.
(DOCX)
