# Supplementary material for: An evaluation model for automobile intelligent cockpit comfort based on improved combination weighting-cloud model
Source: PLoS One. 2023 Mar 3;18(3):e0282602. doi: 10.1371/journal.pone.0282602 (PMC9983905; doi:10.1371/journal.pone.0282602)
Supplement: S1 Table — The table contains the judgment matrix of the second-class indexes C1−C3. It is used to obtain the corresponding second-class weights. (DOCX) [file pone.0282602.s001.docx]

**S1 Table. The** **judgment matrix data of noise and vibration.**

|  | $\boldsymbol{C}_{\boldsymbol{1}}$ | $\boldsymbol{C}_{\boldsymbol{2}}$ | $\boldsymbol{C}_{\boldsymbol{3}}$ |
| --- | --- | --- | --- |
| $\boldsymbol{C}_{\boldsymbol{1}}$ | 1 | 0.650/0.350 | 0.588/0.412 |
| $\boldsymbol{C}_{\boldsymbol{2}}$ | 0.350/0.650 | 1 | 0.259/0.741 |
| $\boldsymbol{C}_{\boldsymbol{3}}$ | 0.412/0.588 | 0.741/0.259 | 1 |

The table contains the judgment matrix of the second-class indexes$C_{1}-C_{3}$. It is used to obtain the corresponding second-class weights.
