# Supplementary material for: An evaluation model for automobile intelligent cockpit comfort based on improved combination weighting-cloud model
Source: PLoS One. 2023 Mar 3;18(3):e0282602. doi: 10.1371/journal.pone.0282602 (PMC9983905; doi:10.1371/journal.pone.0282602)
Supplement: S2 Table — The table contains the judgment matrix of the second-class indexes C4−C6. It is used to obtain the corresponding second-class weights. (DOCX) [file pone.0282602.s002.docx]

**S2 Table.** **The judgment matrix data of light environment.**

|  | $\boldsymbol{C}_{\mathbf{4}}$ | $\boldsymbol{C}_{\mathbf{5}}$ | $\boldsymbol{C}_{\mathbf{6}}$ |
| --- | --- | --- | --- |
| $\boldsymbol{C}_{\mathbf{4}}$ | 1 | 0.412/0.588 | 0.231/0.769 |
| $\boldsymbol{C}_{\mathbf{5}}$ | 0.588/0.412 | 1 | 0.556/0.444 |
| $\boldsymbol{C}_{\mathbf{6}}$ | 0.769/0.231 | 0.444/0.556 | 1 |

The table contains the judgment matrix of the second-class indexes$C_{4}-C_{6}$. It is used to obtain the corresponding second-class weights.
