# Supplementary material for: An evaluation model for automobile intelligent cockpit comfort based on improved combination weighting-cloud model
Source: PLoS One. 2023 Mar 3;18(3):e0282602. doi: 10.1371/journal.pone.0282602 (PMC9983905; doi:10.1371/journal.pone.0282602)
Supplement: S3 Table — The table contains the judgment matrix of the second-class indexes C7−C10. It is used to obtain the corresponding second-class weights. (DOCX) [file pone.0282602.s003.docx]

**S3 Table. The judgment matrix data of thermal environment.**

|  | $\boldsymbol{C}_{\mathbf{7}}$ | $\boldsymbol{C}_{\mathbf{8}}$ | $\boldsymbol{C}_{\mathbf{9}}$ | $\boldsymbol{C}_{\mathbf{10}}$ |
| --- | --- | --- | --- | --- |
| $\boldsymbol{C}_{\mathbf{7}}$ | 1 | 0.571/0.429 | 0.625/0.375 | 0.625/0.375 |
| $\boldsymbol{C}_{\mathbf{8}}$ | 0.429/0.571 | 1 | 0.556/0.444 | 0.394/0.606 |
| $\boldsymbol{C}_{\mathbf{9}}$ | 0.375/0.625 | 0.444/0.556 | 1 | 0.412/0.588 |
| $\boldsymbol{C}_{\mathbf{10}}$ | 0.375/0.625 | 0.606/0.394 | 0.588/0.412 | 1 |

The table contains the judgment matrix of the second-class indexes$C_{7}-C_{10}$. It is used to obtain the corresponding second-class weights.
