# Supplementary material for: An evaluation model for automobile intelligent cockpit comfort based on improved combination weighting-cloud model
Source: PLoS One. 2023 Mar 3;18(3):e0282602. doi: 10.1371/journal.pone.0282602 (PMC9983905; doi:10.1371/journal.pone.0282602)
Supplement: S4 Table — The table contains the judgment matrix of the second-class indexes C11−C15. It is used to obtain the corresponding second-class weights. (DOCX) [file pone.0282602.s004.docx]

**S4 Table. The judgment matrix data of human-computer interaction.**

|  | $\boldsymbol{C}_{\mathbf{11}}$ | $\boldsymbol{C}_{\mathbf{12}}$ | $\boldsymbol{C}_{\mathbf{13}}$ | $\boldsymbol{C}_{\mathbf{14}}$ | $\boldsymbol{C}_{\mathbf{15}}$ |
| --- | --- | --- | --- | --- | --- |
| $\boldsymbol{C}_{\mathbf{11}}$ | 1 | 0.625/0.375 | 0.588/0.412 | 0.606/0.394 | 0.714/0.286 |
| $\boldsymbol{C}_{\mathbf{12}}$ | 0.375/0.625 | 1 | 0.412/0.588 | 0.588/0.412 | 0.588/0.412 |
| $\boldsymbol{C}_{\mathbf{13}}$ | 0.412/0.588 | 0.588/0.412 | 1 | 0.541/0.459 | 0.690/0.310 |
| $\boldsymbol{C}_{\mathbf{14}}$ | 0.394/0.606 | 0.412/0.588 | 0.459/0.541 | 1 | 0.667/0.333 |
| $\boldsymbol{C}_{\mathbf{15}}$ | 0.286/0.714 | 0.412/0.588 | 0.310/0.690 | 0.333/0.667 | 1 |

The table contains the judgment matrix of the second-class indexes$C_{11}-C_{15}$. It is used to obtain the corresponding second-class weights.
