# Supplementary material for: An evaluation model for automobile intelligent cockpit comfort based on improved combination weighting-cloud model
Source: PLoS One. 2023 Mar 3;18(3):e0282602. doi: 10.1371/journal.pone.0282602 (PMC9983905; doi:10.1371/journal.pone.0282602)
Supplement: S5 Table — The table contains the judgment matrix of the first-class indexes B1−B4. It is used to obtain the corresponding first-class weights. (DOCX) [file pone.0282602.s005.docx]

**S5 Table. The judgment matrix data of first-class index.**

|  | $\boldsymbol{B}_{\boldsymbol{1}}$ | $\boldsymbol{B}_{\boldsymbol{2}}$ | $\boldsymbol{B}_{\boldsymbol{3}}$ | $\boldsymbol{B}_{\boldsymbol{4}}$ |
| --- | --- | --- | --- | --- |
| $\boldsymbol{B}_{\boldsymbol{1}}$ | 1 | 0.523/0.477 | 0.421/0.579 | 0.434/0.566 |
| $\boldsymbol{B}_{\boldsymbol{2}}$ | 0.477/0.523 | 1 | 0.347/0.653 | 0.377/0.623 |
| $\boldsymbol{B}_{\boldsymbol{3}}$ | 0.579/0.421 | 0.653/0.347 | 1 | 0.447/0.553 |
| $\boldsymbol{B}_{\boldsymbol{4}}$ | 0.566/0.434 | 0.623/0.377 | 0.553/0.447 | 1 |

The table contains the judgment matrix of the first-class indexes$B_{1}-B_{4}$. It is used to obtain the corresponding first-class weights.
